# Pyoderma gangrenosum‐like lesions in the setting of IgA cutaneous vasculitis: Favourable response to adalimumab

**DOI:** 10.1002/ski2.347

**Published:** 2024-02-06

**Authors:** Hitomi Sugino, Hikaru Kawahara, Kayo Yamamoto, Etsuko Okada, Yu Sawada

**Affiliations:** ^1^ Department of Dermatology University of Occupational and Environmental Health Kitakyushu Japan

## Abstract

Pyoderma gangrenosum is a rare inflammatory skin disease classified within the group of neutrophilic dermatoses, and clinically characterised by painful, rapidly evolving cutaneous ulcers with undermined, irregular, erythematous‐violaceous edges. Underlying diseases include rheumatoid arthritis, inflammatory bowel disease, haematopoietic malignancy, and aortitis syndrome. However, there was a limited number of cases of concomitant pyoderma gangrenosum and IgA vasculitis. Herein, we report a case presenting persistent large skin wounds as a diagnosis of pyoderma gangrenosum in the setting of IgA cutaneous vasculitis, which was successfully treated by a TNF‐α inhibitor. A 67‐year‐old obese female presented palpable purpura on her lower extremities. A skin biopsy taken from the purpuric eruption showed leukocytoclastic vasculitis with IgA and C3 depositions in the vessel walls of the upper dermis, leading to the diagnosis of IgA vasculitis. Small skin ulcers rapidly expanded in several days, eventually developing perforating skin ulcers with irregular erythematous and violaceous edges on both lower extremities following the tapered oral prednisolone at a dose of 25 mg per day. Based on the clinical manifestation and histological analysis, we diagnosed her skin wound as pyoderma gangrenosum. After the adalimumab administration, the spreading ulceration was dampened, leading to the acceleration of wound epithelialisation.

## Graphical abstract

Pyoderma gangrenosum is a rare inflammatory skin disease classified within the group of neutrophilic dermatoses, and clinically characterised by painful, rapidly evolving cutaneous ulcers with undermined, irregular, erythematous‐violaceous edges. Underlying diseases include rheumatoid arthritis, inflammatory bowel disease, haematopoietic malignancy, and aortitis syndrome. Herein, we report a case presenting persistent large skin wounds as a diagnosis of pyoderma gangrenosum following IgA vasculitis, which was successfully treated by a TNF‐α inhibitor.

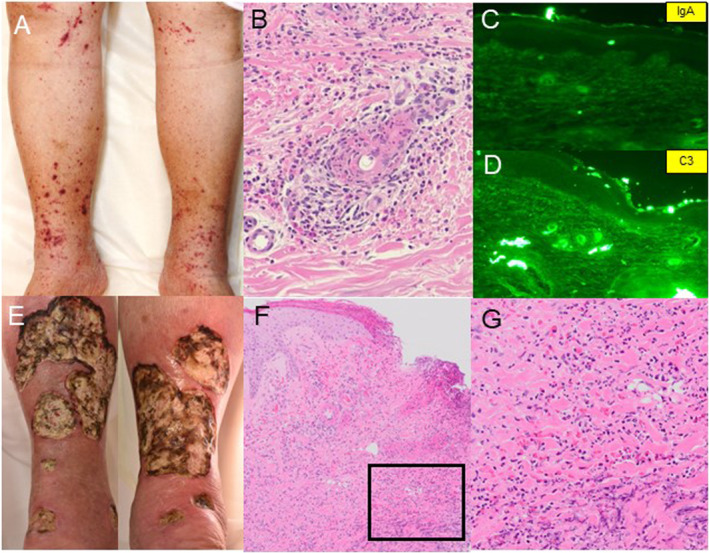

## 1 INTRODUCTION

Pyoderma gangrenosum is a rare inflammatory skin disease classified within the group of neutrophilic dermatoses, and clinically characterised by painful, rapidly evolving cutaneous ulcers with undermined, irregular, erythematous‐violaceous edges. Underlying diseases include rheumatoid arthritis, inflammatory bowel disease, haematopoietic malignancy, and aortitis syndrome.^1^ However, there was a limited number of cases of concomitant pyoderma gangrenosum and IgA vasculitis. Herein, we report a case presenting persistent large skin wounds as a diagnosis of pyoderma gangrenosum in the setting of IgA cutaneous vasculitis, which was successfully treated by a TNF‐α inhibitor.

**FIGURE 1:**
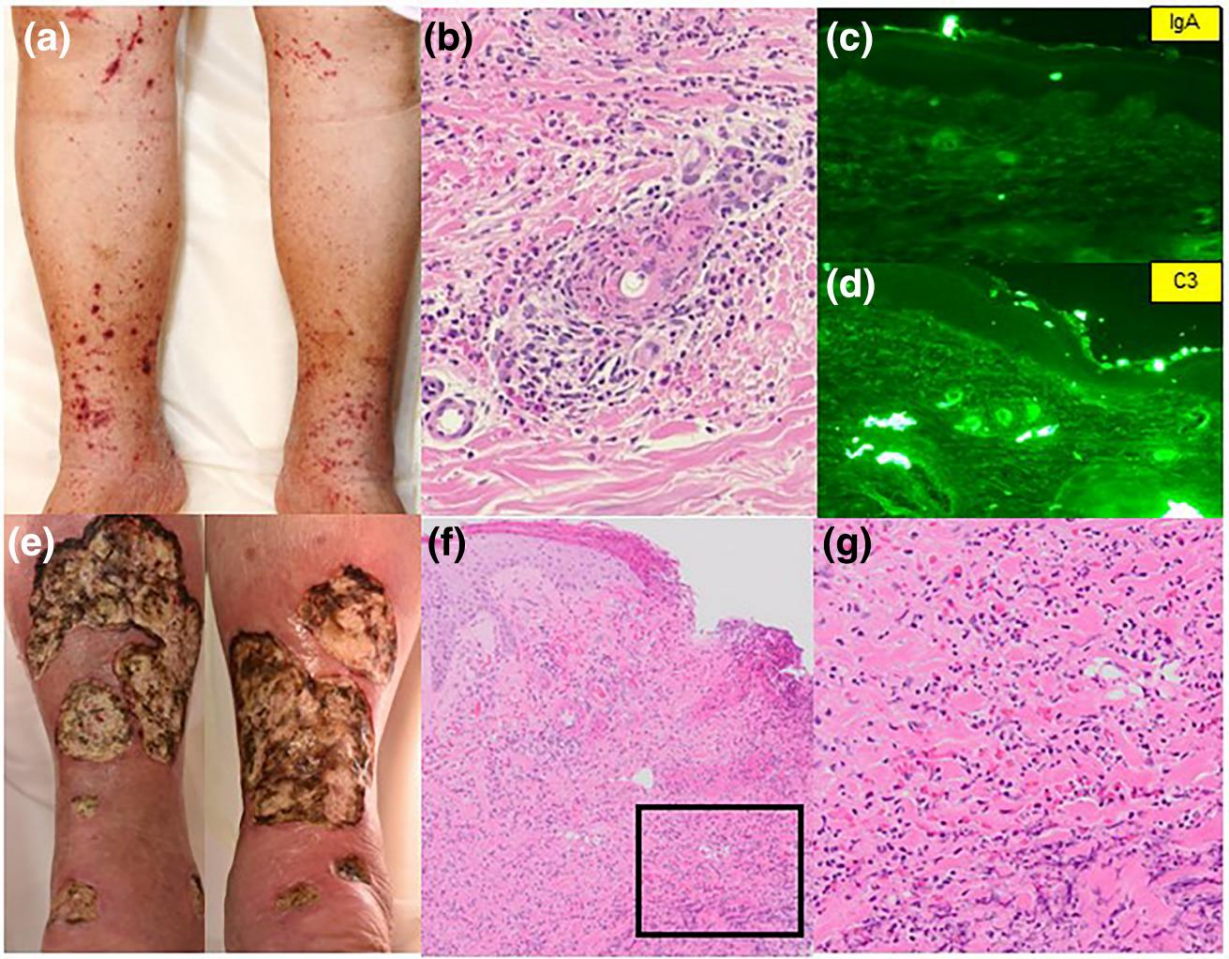
Clinical manifestations and histological analysis. (a) Clinical manifestation. Numerous palpable purpuras ranged in size from a mother's fingertip to a millet grain were distributed in the thigh and dorsum of the foot. (b–d) Histological analyses. (b) H&E staining. (c, d) Direct immunofluorescence of IgA and C3. (e) Clinical manifestation. Perforating skin ulcers with irregular erythematous and violaceous edges were developed on both lower extremities. (f, g) H&E staining.

A 67‐year‐old obese female with the diagnosis of Gorlin syndrome presented palpable purpura on her lower extremities. She visited our hospital for the evaluation of her spreading purpuric skin eruption. She was prescribed eldecalcitol for osteoporosis and olmesartan, spironolactone, and azosemide for hypertension. A physical examination showed that numerous palpable purpuras ranging in size from a mother's fingertip to a millet grain were distributed in the thigh and dorsum of the foot (Figure 1a). Neither gastrointestinal symptoms nor joint dysfunction were present. A skin biopsy taken from the purpuric eruption showed leukocytoclastic vasculitis with IgA and C3 depositions in the vessel walls of the upper dermis with the negative results of MPO‐ANCA and PR3‐ANCA (Figure 1b–d). From these findings, we diagnosed her eruption as IgA vasculitis. After the administration of oral prednisolone 40 mg/day and colchicine, her skin eruption was improved without recurrence of purpura. However, small skin ulcers rapidly expanded in several days, eventually developing perforating skin ulcers with irregular erythematous and violaceous edges on both lower extremities following the tapered oral prednisolone at a dose of 25 mg per day (Figure 1e). An additional skin biopsy showed infiltration of neutrophils throughout the dermal without deposition of IgA and C3 in the vessel walls (Figure 1f,g). Based on the clinical manifestation and histological analysis, we diagnosed her skin wound as pyoderma gangrenosum. She received a colon fibre examination test and a colon tissue biopsy, and both of which denied the existence of inflammatory bowel disease as described previously.^2^ Because the wound was intractable by the treatment of oral corticosteroid, adalimumab was administrated. After the adalimumab administration, the spreading ulceration was dampened, leading to the acceleration of wound epithelialisation.

The concomitant of pyoderma gangrenosum and IgA vasculitis is currently an entirely rare case which is absolutely four cases recognized only in Japan (Table S1).^3^, ^4^, ^5^ IgA is enabled to be involved in both pathogeneses of PG and IgA vasculitis mediated by neutrophil‐mediated immunological reactions. Therefore, the rarity might be underestimated because of the unrecognized aetiology for dermatologists.

TNF‐α inhibitors induce various adverse effects including vasculitis, thromboembolic events, and other autoimmune diseases. Inflammatory bowel diseases are sometimes complicated by IgA vasculitis following TNF‐α inhibitors therapy mediated by the deposition of TNF‐α inhibitor and TNF‐α immune complexes in vessels leading to direct drug toxicity, autoantibody production, and a shift of the Th2 dominant immune profile.^6^ However, our case clearly provided the efficacy of TNF‐α inhibitor, indicating that TNF‐α inhibitor might be the candidate therapeutic option against intractable skin wounds due to pyoderma gangrenosum in the setting of IgA cutaneous vasculitis.

## CONFLICT OF INTEREST STATEMENT

None to declare.

## AUTHOR CONTRIBUTIONS

**Hitomi Sugino**: Conceptualization (lead); data curation (lead); formal analysis (lead); funding acquisition (lead); investigation (lead); methodology (lead); project administration (lead); resources (lead); software (lead); writing – original draft (lead); writing – review & editing (lead). **Hikaru Kawahara**: Conceptualization (equal); formal analysis (equal); writing – review & editing (equal). **Kayo Yamamoto**: Conceptualization (equal); data curation (equal); formal analysis (equal); writing – original draft (equal). **Etsuko Okada**: Conceptualization (equal); data curation (equal); formal analysis (equal); investigation (equal); methodology (equal); project administration (equal); writing – review & editing (equal). **Yu Sawada**: Investigation (equal); methodology (equal); project administration (equal); supervision (equal); validation (equal); writing – original draft (lead); writing – review & editing (lead).

## ETHICS STATEMENT

Not applicable.

## Supporting information

Table S1

## Data Availability

Data sharing is not applicable to this article as no new data were created or analyzed in this study.
