# Supplementary material for: Pyoderma gangrenosum‐like lesions in the setting of IgA cutaneous vasculitis: Favourable response to adalimumab
Source: Skin Health Dis. 2024 Feb 6;4(2):e347. doi: 10.1002/ski2.347 (PMC10988711; doi:10.1002/ski2.347)
Supplement: Supplementary file 1 — Table S1 [file SKI2-4-e347-s001.docx]

| Supplementary Table 1. Literature review of the cases of IgA vasculitis and pyoderma gangrenosum | | | | | | |
| --- | --- | --- | --- | --- | --- | --- |
| Authors | **Age/**  **Sex** | **Pyoderma gangrenosum** | **Pathology**  **(PG)** | **Symptoms of IgA vasculitis** | **Trigger** | **Therapy** |
| Murayama N, et al | 37/  Male | Abdomen, lower leg | Vasculitis  (-) | Purpura of the lower leg,  joint pain, muscle pain | Streptococcal infection | PSL3mg/day |
| Kobayashi M, et al. | 18/  Male | Cheek | Vasculitis  (-) | Purpura of the lower leg, abdominal pain | Trauma | PSL40mg/day |
| Ito M, et al. | 29/  Male | Lower jaw | Vasculitis  (+) | Purpura of the lower leg,  joint pain, abdominal pain | Incision？ | PSL40mg/day  →mPSL1g/day 3days |
| Our case | 67/  Female | Lower legs | Vasculitis  (-) | Purpura of the lower leg,  urinary occult blood | Trauma | PSL40mg/day  →Adalimumab |
